# Political Ideology and Trust in Government to Ensure Vaccine Safety: Using a U.S. Survey to Explore the Role of Political Trust

**DOI:** 10.3390/ijerph20054459

**Published:** 2023-03-02

**Authors:** Jaeyoung Lim, Kuk-Kyoung Moon

**Affiliations:** 1 Department of Public Administration and Social Welfare, Chosun University, Gwangju 61452, Republic of Korea; 2 Department of Public Administration, Inha University, Incheon 22212, Republic of Korea

**Keywords:** political trust, political ideology, government ensuring vaccine safety

## Abstract

Since early 2020, the rapid expansion of COVID-19 has raised concerns about vaccine safety and the government’s handling of it. Particularly notable and concerning has been a growing number of people who oppose vaccines, as this opposition poses a threat to public health. Those for and against vaccination have become polarized along a political divide. Within this context, this study focuses on the role of political trust, exploring whether political ideology is associated with the perception that the government can ensure the safety of vaccines and whether there is a moderator that can alleviate the concerns of those who oppose the government’s handling of vaccine safety on ideological grounds. This study relies on the 2021 U.S. General Social Survey (GSS) and employs an ordered probit method because the dependent variable is an ordered category. The ordered probit model includes a weight provided by the U.S. GSS to account for the population. The sample size was 473 because of the inclusion of all the variables relevant to this study. The results obtained are as follows: First, conservatives associate negatively with support for the government’s handling of vaccine safety. Second, more importantly, conservatives exhibit a higher trust level toward the government to ensure vaccine safety if their level of political trust increases. The results point to important implications. Political ideology matters in how individuals view the government’s handling of vaccine safety. Political trust plays a key role in helping individuals alter their views toward the government’s handling of vaccine safety. This points to a need for the government to take political trust seriously and work hard to improve the public’s trust in the government.

## 1. Introduction

The spread of COVID-19 since 2020 has upended hundreds of millions of lives around the world. As of 13 February 2023, over 6.8 million lives around the world have been lost because of the global coronavirus outbreak [1]. The virus has forced governments and companies to produce vaccines. Government-supported programs, such as Operation Warp Speed in the U.S., have invested billions of dollars into creating a vaccine that could work as a common defense against COVID-19, eventually succeeding in producing multiple effective vaccines [2]. An academic study estimated that without vaccines, over 14 million additional lives would have been lost between late 2020 and late 2021 [3]. As of 30 November 2022, over 25 vaccines have been used around the world, including the well-known vaccines from Pfizer-BioNTech, Oxford-AstraZeneca, Moderna, Novavax, Johnson & Johnson, and Sinopharm [1].

Vaccines have also continued to be improved, to the point that the latest booster shots at the end of December 2022 were found to reduce hospitalization risk approximately 50% more than the original vaccines did [4]. Vaccines have been shown to provide a formidable defense against longer hospitalizations and potential fatalities [4]. As of 13 February 2023, roughly 72.1% of the world population has had at least one dose of a COVID-19 vaccine. However, although a significant number of countries have over 80% full vaccination (having two doses or more), many low-income countries, particularly in Africa, have had difficulty obtaining and administering vaccines, and some developed countries such as the United States experience strong opposition to vaccination despite a wealth of vaccine options [1,3].

Thus, developing vaccines is one thing and improving vaccination rates is another [5]. Despite aggressive efforts by the government to push up the vaccination rate, some individuals have been ideologically adamant in refusing to take it as several surveys have demonstrated [5]. Recently, conservative politicians in the U.S. voted for putting an end to a vaccine mandate for some healthcare workers and to the public health state of emergency declared at the inception of the coronavirus pandemic [6]. Misinformation about the benefits of vaccines and their safety has continued to confound governmental efforts to protect public health from COVID-19 [7]. Within this context, we began to study the extent to which individuals’ political ideology matters in shaping their attitudes toward government-ensured vaccine safety. We also set out to identify factors that may play a role in alleviating individuals’ politically oriented, hostile attitudes toward government-ensured vaccine safety. In doing so, we focused on political trust, as it is considered a foundation for sustaining and strengthening democratic governance [8].

Thus, our study begins with the role of political ideology in explaining the degree to which individuals trust the government to ensure vaccine safety. Political ideology concerns individuals’ attitudes toward politics, and it is naturally implicated in how individuals conceive of the government and its programs. Scholars have noted that political ideology plays an enduring and symbolic role in reshaping individuals’ views toward public policies, as it functions as a guide to processing and organizing information about the details of a given policy issue [9]. For simplicity of political ideology’s complicated concepts, we focus on liberals and conservatives as two major types of individuals with political ideologies [10]. At least in the U.S., conservatives have supported limited government and considered governmental expansion as a threat to individual freedom, whereas liberals, on the other hand, have been more receptive to the government promoting economic and social equality [11]. This stance also applies to public health issues such as vaccination, as several studies have pointed out the connections between individuals’ political leanings and vaccine hesitancy [12,13,14]. The research in this area has demonstrated that liberals are more inclined toward vaccination, while conservatives are more disinclined [12,13,14]. These studies have led to the reasonable conjecture that liberals would show positive attitudes toward the government’s ability to ensure vaccine safety whereas conservatives would show negative attitudes toward it.

Second, our study points to the role of political trust in explaining individuals’ trust in the government to ensure vaccine safety. Scholars have noted that individuals with significant confidence in political institutions view government policies and activities positively [15,16]. The theoretical explanation for this is given in terms of political trust as a heuristic. A heuristic is a psychological shortcut for individuals because they are constrained by resources, information, and capacity and are not well-equipped to assess each government policy or activity rationally [15,16]. Thus, they rely on political trust as a psychological shortcut through which they swiftly either endorse or oppose a government policy or activity [15,16]. Thus, individuals with a low level of political trust are less inclined to support a given policy than those with a high level of political trust [11,15,16,17,18]. Scholars have pointed out that political trust is positively associated with individuals’ views toward public health programs [19,20,21]. Extending this reasoning, we expect that high-trust individuals would view the government’s ability to ensure vaccine safety more positively than low-trust individuals.

More importantly, we focus on the role of political trust as the moderator that helps shape the relationship between conservatives and their level of trust in the government’s role in ensuring vaccine safety. As noted, political trust works as a heuristic that enables individuals to evaluate a government policy or activity without having to accurately analyze it [15,16]. The heuristic function of political trust is not equally applicable to all individuals but falls disproportionately upon some more than others [15,16]. According to the ideological sacrifice theory, political trust’s heuristic effect is magnified when individuals are forced to make a material or ideological sacrifice [15,16]. Because of their ideology, liberals possess a strong penchant for government-led programs and their expansion [15,16]. Thus, they would likely support governmental efforts to ensure vaccine safety, regardless of their present level of political trust. Conservatives significantly differ from liberals because they must sacrifice their ideological preferences to trust the government to ensure vaccine safety [11,15,16,17,18]. Thus, the political trust would work to moderate and weaken the negative relationship between conservatives and their trust in the government to ensure vaccine safety.

Based on these discussions, we focus on examining the following specific questions. First, do individuals’ political ideologies affect their trust in the government to ensure vaccine safety? Second, do individuals’ levels of political trust affect their trust in the government to ensure vaccine safety? Finally, does individuals’ level of trust affect the relationship between their political ideology and their trust in the government to ensure vaccine safety? These research questions, in turn, can be formulated as the following hypotheses for empirical investigation.

**Hypothesis** **1.** *Liberals will be positively associated with trust in the government to ensure vaccine safety*.

**Hypothesis** **2.** *Conservatives will be negatively associated with trust in the government to ensure vaccine safety*.

**Hypothesis** **3.** *Political trust will be positively associated with trust in the government to ensure vaccine safety*.

**Hypothesis** **4.** *Political trust will moderate the relationship between conservatives and trust in the government to ensure vaccine safety. Thus, the degree of the negative relationship between conservatives and trust in the government to ensure safety is reduced as the level of political trust increases*.

The present study makes several contributions to the understanding of political ideology, political trust, and government-handled vaccine programs. First, by exploring the effects of political ideology and political trust on citizens’ perceptions of government-ensured vaccine safety, this study illuminates the role individuals’ political preferences and trust in political institutions play in perceiving how the government handles major public health programs such as ensuring vaccine safety for citizens. Second, the present study makes use of the heuristic function of political trust to illuminate the relationship between individuals’ political ideology and their trust in the government to ensure vaccine safety. In doing so, we offer a sophisticated look at how political trust can be a moderating factor that intervenes in the effect individuals’ political ideology may have on their views of government-ensured vaccine safety. Third, we bring the understudied concept of vaccine safety to this study as its focus. Studies have explored the determining factors of *vaccination willingness* [12,13,14], but few have examined the determining factors of *government-ensured vaccine safety*. Thus, the present study helps diversify vaccine-related concepts and enrich individuals’ attitudes toward them. Lastly, this study examines the above-described aspects using one of the latest datasets widely available for public use: the 2021 U.S. General Social Survey (GSS). In doing, so, we aimed to provide an up-to-date understanding of the dynamic relationships among political ideology, political trust, and government-ensured vaccine safety, as well as an understanding of the government’s pursuit of enhancing public health and safety, such as vaccine safety.

For our study, we have relied on the 2021 U.S. GSS and an ordered probit method because our dependent variable, trust in the government to ensure vaccine safety, is an ordered category. The sample size for our model is 473 because of the inclusion of all the variables relevant to the study. We have also relied on a U.S. GSS-provided weight and Huber—White sandwich estimator to control heteroskedasticity. Furthermore, we have performed a link test [22] and found that our empirical model was not misspecified.

We proceed as follows: First, we examine political ideology and its association with individuals’ trust in the government to ensure vaccine safety. Next, we explore the concept of political trust and its relationship with individuals’ trust in the government to ensure vaccine safety, as well as its potential to moderate the negative relationship between conservatives and trust in the government to ensure vaccine safety. The theoretical explorations will lead us to generate hypotheses and empirically test them. Finally, we will present our empirical results, discuss them, and explore their scholarly and practical implications.

## 2. Materials and Methods

### 2.1. Literature Review and Hypothesis

#### 2.1.1. Political Ideology and Government-Ensured Vaccine Safety

As COVID-19 emerged and spread across borders, it resulted in the deaths of millions of people [23]. Naturally, governments around the world responded with unprecedented efforts to create a vaccine in a short amount of time [23]. Despite the astoundingly successful creation of vaccines, one difficulty remained: raising the vaccination rate. Experts were initially worried about a possible discrepancy among races in terms of the vaccination rate, but active efforts by governments have resulted in boosting vaccinations among racial minorities over time [24]. Still, a gap in vaccination rates has emerged in a politically divided world [25]. A Kaiser Foundation survey conducted on 13–22 September 2021, revealed that 90% of Democrats in the U.S. were vaccinated, whereas only 58% of Republicans were [25]. A Gallup survey released on 29 September 2021, showed a similar trend, with 92% of Democrats in the U.S. and 56% of Republicans vaccinated; 23% of Republicans also expressed that they were determined not to get vaccinated in the future [26]. A survey found that counties voting for Joseph Biden during the 2020 presidential election boasted a higher vaccination rate than those casting their votes for Donald Trump. The evidence points to vaccination refusal being politically motivated [27]. With the virus expected to undergo many variations in the future, it is critical to understand the political divide leading to a vaccination discrepancy because refusal to be vaccinated can trigger adverse consequences for government-led public health measures [5].

What, then, might lead politically motivated individuals to have differing attitudes toward vaccinations? First, some point out the close connection between Republicans’ distrust of science and conservatives’ penchant for not being vaccinated. A Gallup survey found that Republicans’ trust in science has plummeted from 72% in 1975 to 45% today, whereas Democrats’ trust in science has surged from 67% to 79% during the same period [28]. Although most people accept scientific truths, in recent years it has become increasingly mired in politics, with conservatives more likely to distrust scientific facts and the scientific community, which has bleak ramifications for government-led vaccination efforts [29]. Second, others have associated vaccination hesitancy with religious affiliation [13,30]. For instance, some religious groups tend to feel that their worldviews are menaced by science and the idea of evolution by natural selection [13,30]. Third, others have explored the connections between vaccine hesitancy and authoritarianism [13,31]. Individuals tend to identify with authoritarianism when their views are not adequately represented by political elites. When authoritarian leaders sow and propagate doubt with respect to vaccination, individuals sympathetic to authoritarianism also incorporate such skepticism into their views toward vaccination [13], particularly when authoritarianism denotes hierarchical dominance in which the “weak” follow the “strong” [31].

Several studies have examined the connections between individuals’ political leanings and their willingness to be vaccinated [12,13,14,32,33,34,35,36]. Liberals are more likely to support vaccinations, and conservatives are less so. This reasoning can generate a reasonable hypothesis that liberals would be inclined to trust the government to ensure vaccine safety while conservatives distrust it. Thus, we propose the following hypotheses for empirical scrutiny:

***Hypothesis*** ***1.****Liberals will be positively associated with trust in the government to ensure vaccine safety*.

***Hypothesis*** ***2.****Conservatives will be negatively associated with trust in the government to ensure vaccine safety*.

#### 2.1.2. Political Trust and Its Moderation of the Link between Political Ideology and Trust in Government to Ensure Vaccine Safety

Is there a mechanism, then, that would mollify conservatives’ hostile attitudes and lack of trust in the government to ensure vaccine safety? The present study points to political trust as a mechanism through which individuals may support public programs such as government-ensured vaccine safety. Such a role of political trust can be explained by its heuristic function, the impact of which is magnified when individuals have to sacrifice their material or ideological preferences (ideological sacrifice theory) [15,16].

Before we further discuss political trust’s heuristic function and the ideological sacrifice theory, political trust can be defined from three perspectives. First, political trust is based on citizens’ evaluations of government performance [15]. More specifically, political trust refers to individuals’ perceptions of government performance based on their expectations of how the government should function [15,16,37,38]. Second, political trust can also be defined by how individuals see the processes that lead to performance [39]. Third and finally, political trust can refer to government integrity because government corruption often leads to a decline in political trust among citizens [40,41]. The research on political trust was launched in the wake of several political scandals and crises. For example, the political trust of U.S. citizens fell precipitously from over 70% in the 1960s to less than 30% in 1980 [16]. Several events and scandals—the Vietnam War and its repercussions, oil shocks, the Watergate scandal, and more—shattered the confidence of U.S. citizens in political institutions [42,43]. Naturally, in the beginning, studies on political trust centered on what determines political trust [37,39,44]. However, as political trust research evolved, scholars began to pay more attention to its outcomes and moderating effects.

Studies pointing to positive relationships between individuals’ level of political trust and their attitudes toward public programs are centered on the role of political trust as a heuristic [15,16]. Individuals are constrained by a lack of knowledge and resources that would enable them to assess a government policy or activity accurately and quickly [15,16]. Thus, they need an evaluative tool to enable them to do it so swiftly without fully analyzing a given public policy. Political trust is such a tool, being a decisional heuristic by which individuals can evaluate a government policy or activity, even though they are not equipped to analyze it competently and rationally [16]. Because of political trust’s heuristic function, individuals who may not know the full details of a given public policy or are not capable of analyzing them can support the policy. Naturally, individuals who have a high level of trust are likely to give the benefit of doubt to a government-led program or initiative such as public health program [19,20,21].

However, the ideological sacrifice theory dictates that political trust’s positive impact can be magnified when it works for individuals who would sacrifice more because of their material or ideological preferences [16]. In other words, political trust is not a salient tool for all people [16]. For instance, liberals are likely to support social welfare programs, regardless of whether their political trust level is high [17]. In contrast, conservatives are ideologically disinclined to support such programs; they need a high level of political trust to help them make their ideological sacrifices and support such programs [11,16]. For policies such as tax cuts, the opposite is true. Liberals are ideologically inclined to oppose tax cuts and need a high level of political trust to reverse their position because such programs demand ideological sacrifice from them. Conservatives, on the other hand, are ideologically inclined to support tax cuts, so a high level of political trust is required to oppose them [11,16,18]. Put shortly, according to the ideological sacrifice theory, even if conservatives do not like a public policy, they may be willing to sacrifice their ideological preferences and support it if they have a high level of political trust.

Thus, we have theoretical mechanisms—political trust’s heuristic function and the ideological sacrifice theory—to explain why conservatives may support government-ensured vaccine safety if their level of political trust is high. Vaccination has become a topic of heated political discourse between liberals and conservatives. For that reason, for conservatives to trust the government to ensure vaccine safety would go against their ideological preferences and demand ideological sacrifice. Following the reasoning laid out above, the political trust would serve as a heuristic that would help individuals positively assess the government’s trustworthiness to ensure vaccine safety. Additionally, it is also plausible that conservatives would be disinclined to support government programs to ensure vaccine safety, but they would be willing to sacrifice their ideological preferences and support them if moderated by political trust. Based on the discussion so far, we propose the following hypotheses for empirical examination:

***Hypothesis*** ***3.****Political trust will be positively associated with trust in the government to ensure vaccine safety*.

***Hypothesis*** ***4.****Political trust will moderate the relationship between conservatives and trust in the government to ensure vaccine safety. Thus, the degree of the negative relationship between conservatives and trust in the government to ensure safety is reduced as the level of political trust increases*.

### 2.2. Data Management

We relied on the U.S. General Social Survey (GSS) for the empirical analysis. Having been administered by NORC, Chicago University, since 1972, the U.S. GSS is cross-sectional in nature, and each survey is centered on a replicated set of items, along with a set of topical items that may not be repeated [45]. Although the U.S. GSS has been conducted primarily in person, the 2021 U.S. GSS was collected mainly through a web portal because of safety concerns caused by the COVID-19 pandemic [45]. The U.S. GSS employs full-probability sampling based on the adaptation of the United States Postal Service’s metropolitan area [45]. For the empirical analysis of the model explored in the present study, we relied on the 2021 U.S. GSS version, which yielded 473 observations because of the inclusion of all variables relevant to our study.

### 2.3. Variables

#### 2.3.1. Trust in Government to Ensure Vaccine Safety

The dependent variable of our model is trust in the government to ensure vaccine safety. Here, one item consists of ordinal values ranging from 1 to 3. The respondents were asked how much confidence they place in the federal government to ensure vaccine safety to “protect the public against serious diseases” [45] (p. 143). The item was reverse-coded so that 3 indicates *a great deal* of confidence, 2 indicates *only some*, and 1 indicates *hardly any* [34].

#### 2.3.2. Political Ideology

In our model, the political ideology variables are liberals and conservatives, both of which are indicator variables. The two variables are derived from one item through which the respondents were asked to indicate their political preferences on a 7-point scale: 1 (*extremely liberal*), 2 (*liberal*), 3 (*slightly liberal*), 4 (*moderate/middle of the road*), 5 (*slightly conservative*), 6 (*conservative*), and 7 (*extremely conservative*) [45]. Values ranging from 1 to 3 were coded as liberal, and those ranging from 5 to 7 were coded as conservative. A value of 4 was coded as moderate and dropped from the model because it served as the reference variable. As hypothesized earlier, we expected that the class identifying as liberal would likely be positively associated with trust in the government to ensure vaccine safety (Hypothesis 1) and that the class identifying as conservative would likely be negatively associated with it (Hypothesis 2).

#### 2.3.3. Political Trust

Political trust served as the moderator; it was measured as the summed average of two items. The respondents were asked to indicate their confidence in the federal government (3-point scale) and Congress (3-point scale) [45]. The correlation between the two items was 0.49. The two items were coded reversely so that a value of 3 indicated *a great deal* of confidence and a value of 1 indicated *hardly any* confidence. As hypothesized earlier, we expected that political trust would be positively associated with trust in the government to ensure vaccine safety (Hypothesis 3) and would moderate the negative relationship between conservatives and trust in the government to ensure vaccine safety because conservatives, contingent upon the level of political trust, may be willing to make ideological sacrifices in trusting the government to ensure vaccine safety (Hypothesis 4).

#### 2.3.4. Control Variables

We also accounted for a group of control variables. First, in our model, we included distrust in the scientific community. The respondents were asked to indicate their level of trust in the scientific community. A value of 3 indicated *hardly any* trust in the scientific community, and a value of 1 indicated *a great deal* of trust [45]. Scholars have noted that individuals with a high degree of distrust in science are less likely to support the government and its handling of scientific programs [29,46]. Because science is at the heart of vaccine-related matters, we expected a negative relationship between distrust in the scientific community and trust in the government to ensure vaccine safety.

Second, we also accounted for the respondents’ demographic variables: age, female, white, education, and income. Female (coded as 1) was an indicator variable, and the reference variable dropped (coded as 0) was male. White (coded as 1) was an indicator variable, and the reference variable omitted from the model was nonwhite respondents. Education and income were measured as levels. Education was measured as the years of education received: for example, 0 denoted *no formal education*, 12 denoted *completion of high school*, and 20 denoted *8 years of college education*. Income referred to the previous year’s total family income from all sources. For instance, 1 denoted *under USD 1000*; 10 denoted *USD 12,500–USD 14,999*; 20 denoted *USD 60,000–USD 74,999*; and 26 was the highest level of income measured, *USD 170,000 or over*. Finally, age was measured in years.

Table 1 displays the descriptive statistics of the variables in the model. The mean of the dependent variable was 2.27 out of the 1–3 range, indicating that respondents were on average more trusting than distrusting of the federal government’s handling of vaccine safety. In terms of political ideology, roughly 36% of the respondents identified themselves as liberals, and roughly 33% considered themselves conservatives. Thus, the percentage of liberals was slightly higher than that of conservatives in the dataset. The mean of political trust was 1.55, suggesting that the respondents were, on average, more distrusting than trusting political institutions. The mean of distrust in the scientific community was 1.54, indicating that respondents place more trust than distrust in the scientific community. The mean age was 52.53 years; the percentage of female respondents was 54%; that of white respondents was 82%; the educational level of respondents on average was 14.88 (close to 15, 3 years of education including college); the mean level of income was 18.51 between level 18 (USD 40,000 to USD 49,999) and level 19 (USD 50,000 to USD 59,999).

Table 2 displays Pearson correlations between the variables used for the model. Liberal identification (r = 0.13, *p* < 0.01) is positively correlated with trust in the government to ensure vaccine safety, whereas conservative identification (r = −0.16, *p* < 0.01) is negatively correlated with the dependent variable. Political trust (r = 0.30, *p* < 0.01) is positively correlated with the dependent variable. On the contrary, a greater level of distrust in the scientific community (r = −0.35, *p* < 0.01) is negatively correlated with the dependent variable. Greater age (r = 0.15, *p* < 0.01) is positively correlated with trust in the government to ensure vaccine safety. Similarly, a higher level of education (r = 0.13, *p* < 0.01) has a positive relationship with the dependent variable. Women (r = −0.11, *p* < 0.01) display negative relationships with trust in the government to ensure vaccine safety. Finally, race and income are not significantly correlated with the dependent variable.

## 3. Results

For our study, we relied on the 2021 U.S. GSS and an ordered probit method through the statistical software Stata 14 because our dependent variable—trust in the government to ensure vaccine safety—consisted of ordinal values. We also accounted for the U.S. GSS-provided weight and Huber—White sandwich estimator to control heteroskedasticity. Additionally, we performed a link test to see if our model was misspecified and found that our empirical model is specified correctly; based on the link test, the prediction squared showed no explanatory power [22]. A confirmatory factor analysis was not conducted due to the fact that our model had only one variable (political trust) consisting of two items or more and every other variable was based on one item. As noted earlier, political trust consists of two items and we performed a principal component factor analysis and found that the eigenvalue for the two items forming political trust was 1.50, greater than the commonly accepted threshold of 1.00.

Table 3 shows hierarchical regression analyses that consist of two models. In Model 1, we focused on the direct relationships between the independent and dependent variables. In Model 2, we examined the interaction term—Conservative × Political Trust—which explores the moderation of political trust of the negative relationship between conservatism and trust in the government to ensure vaccine safety. Wald $\chi^{2}$ test statistics for both models were 88.11 and 92.64, respectively, and their *p*-values were <0.01, indicating that all the coefficients in both models were nonzero.

The results confirmed three hypotheses (Hypotheses 2–4) and failed to confirm one (Hypothesis 1). First, in terms of direct relationships (Model 1), Hypothesis 2 was confirmed. Conservatism was negatively associated with trust in the government to ensure vaccine safety (*β* = −0.19, *p* < 0.05). Conservatives were more likely to show antagonism toward governmental activities and programs, and they were less likely to approve and embrace vaccination and the government’s assurance of vaccine safety. Thus, because of these ideological preferences, conservatives were not inclined to trust the government to ensure vaccine safety. The results, however, failed to confirm Hypothesis 1 that there would be a positive link between liberals and trust in the government to ensure vaccine safety. Because “moderates” serve as the reference variable, it can be surmised that liberals do not significantly deviate from moderates in their attitudes toward the government’s ensuring vaccine safety.

Additionally, Hypothesis 3 was confirmed. Political trust was positively associated with trust in the government to ensure vaccine safety (*β* = 0.45, *p* < 0.01). Because political trust is a heuristic that enables individuals to judge governmental programs or activities positively when they do not have the time, resources, or capacity to analyze them accurately, we can conjecture that individuals with a high degree of political trust may exhibit trust in the government to ensure vaccine safety.

More importantly, the present study centered on the role that political trust may play in moderating and dampening the negative relationship between conservatism and trust in the government to ensure vaccine safety. The results from Model 2 confirmed Hypothesis 4, pointing to the role of political trust in altering conservatives’ attitudes (*β* = 0.32, *p* < 0.05). As noted earlier, political trust is a vital heuristic through which individuals may favorably view government programs or activities. This heuristic function also works for individuals who may have to sacrifice their material or ideological interests. In other words, individuals may be willing to risk their material well-being or ideological preferences to support the government and its programs if they possess a high level of political trust. The results of the model clearly show that, equipped with a high degree of political trust, conservatives who would otherwise be opposed to government programs such as ensuring vaccine safety may indeed support it. Thus, the results confirmed the capacity of political trust as a heuristic to help conservatives overcome their ideological or material barriers with respect to a given governmental policy or activity.

Finally, in terms of the control variables, two variables were significantly associated with the dependent variable. As expected, a high degree of distrust in the scientific community was negatively associated with trust in the government to ensure vaccine safety (*β* = −0.64, *p* < 0.01). Because individuals in this class are not likely to trust vaccination and its benefits [18,35], they cast skepticism on government vaccination operations and programs to ensure its safety. Additionally, the results show a positive association between age and trust in the government to ensure vaccine safety (*β* = 0.02, *p* < 0.01). As individuals grow older, they feel vulnerable to various diseases and approach vaccination with more eagerness and approval than those who are younger; they are also likely to be prioritized by the government for vaccination because of their weaker immune systems [36]. Other demographic variables, at least in our dataset, were not shown to be significantly associated with trust in the government to ensure vaccine safety.

**Figure 1:**
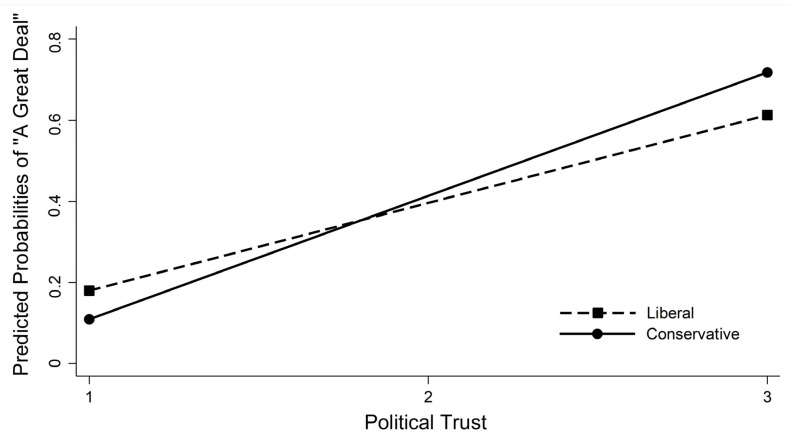
Predicted probabilities of support for “a great deal” of trust in government to ensure vaccine safety.

Figure 1 shows the moderating effects of political trust (where the value of the dependent variable is 3, *a great deal of trust*) on the negative relationship between conservatives and trust in the government to ensure vaccine safety. The solid line denotes the linkage between conservative identification and *a great deal of trust* when the level of political trust increases; the dashed line refers to the linkage between liberal identification and *a great deal of trust* when the level of political trust increases.

As can be seen, the slope of both the solid and dashed lines is positive; however, the slope of the solid line is steeper than that of the dashed line. The degree of strong trust the in government to ensure vaccine safety among both liberals and conservatives increases as their level of political trust increases. However, conservatives’ degree of strong trust (the dependent variable) is greater than that of liberals as the level of political trust increases. The graph and the results noted in Table 3 clearly show that the heuristic function of political trust does not operate equally for liberals and conservatives. Rather, its impact is more palpable for conservatives because they risk their ideological preferences when they trust the government to ensure vaccine safety.

## 4. Discussion

The results suggest several implications for policymakers. First, ideology matters in contemporary discussions of vaccination and governmental involvement in its safety. In our model, conservatism was negatively associated with trust in the government to ensure vaccine safety. The results confirmed several studies that have pointed out the negative connections between conservatism and vaccination [13,14,33,35,47]. Moreover, ideology has become more intransigent than ever before, with political polarization resulting in an ever-widening gulf between liberals and conservatives [48,49]. Ideological polarization raises an urgent need for improving the level of public trust in the government, particularly for a government intent on improving vaccination rates among the public and public perceptions of the government’s vaccination management.

The results also showed that liberalism was not significantly associated with trust in the government to ensure vaccine safety, whereas conservatism was significantly and negatively associated with the dependent variable. Because moderates served as the reference variable and were omitted from the model, a few reasonable conjectures can be made. First, liberals are not distinguishable from moderates when it comes to trusting the government to ensure vaccine safety. In fact, the correlation between liberal ideology and the dependent variable was 0.13, that between moderate ideology and the dependent variable was 0.02, and that between conservative ideology and the dependent variable was −0.16. Thus, there was a larger discrepancy between moderates and conservatives in their relation to the dependent variable than between moderates and liberals. This partly explains why liberalism did not significantly correlate with trust in the government to ensure vaccine safety.

The results of the model also pointed out that liberalism’s relation to the dependent variable was not moderated by the level of political trust. As mentioned earlier, liberals are, in general, more supportive of a government policy or activity than conservatives [15,16,38]. It is possible for political trust to further strengthen such attitudes by liberals, but political trust is not an absolute necessity for liberals in their support of the government. Conservatives are ideologically predisposed to dislike expanded governmental reach [15,16,38]. For conservatives, vaccination is a personal choice and, therefore, is not a matter of government mandates [50]. Because they need to make some ideological sacrifice to support governmental involvement in vaccination and its safety, a high dose of political trust needs to be activated before conservatives will place trust in the government to ensure vaccine safety. This is why the results did not show the moderation effect of political trust on the linkage between liberals and the dependent variable, while conservatives’ attitudes toward trust in the government ensuring safety were altered by political trust.

The results of the present study point to the following implications for public officials to improve the level of public trust in the government. First, citizens need to have their voices heard by the government. According to an OECD survey, 47.8% of global respondents believed they could meaningfully engage in politics [8]. When citizens believe they can be change agents in democratic political processes, they are more likely to have a greater level of confidence in the government. Positive feedback from political participation reinforces citizens’ political trust by adding legitimacy to the political system [Putnam, 2000]; a low level of political participation is associated with low levels of political trust [Parvin, 2018]. Thus, public officials need to identify channels through which citizens can raise their voices and meaningfully participate in political processes such as holding public forums and making citizen participation easy through digital transformation.

Second, the government needs to conduct public affairs free of corruption to raise the political trust level of citizens. Maintaining and fostering the integrity of public offices is crucial to gain public confidence in political institutions. There is a close connection between political distrust of citizens and political corruption in public offices [29,30]. Third, the government needs to function competently and raise its performance in conducting public affairs, as there is a positive connection between government performance and citizens’ political trust [6]. In the early days of the COVID-19 pandemic, the U.S. federal government suffered from political distrust because of its lack of responsiveness to the emerging crisis; there were problems such as failures to develop and secure test kits, implement vaccinations, improve vaccination rates, and coordinate efficient state and national responses [42]. Improving government performance would involve improving the efficiency of government work, implementing policies that are just and equitable, reflecting public input into political and public affairs, managing economic and natural crises effectively and efficiently, strengthening government capacity to prepare for unforeseen events, and more.

Fourth, governments should pay attention to how to deliver government messages to the public. Citizens are increasingly dependent on social media to obtain information on public policies, but the government is not utilizing social media as a major platform for providing government information to the public [8]. Misinformation about vaccines and their safety has greatly frustrated both government officials and healthcare providers, leading to overwhelmed hospitals, prolonged hospitalizations, and even premature deaths [7]. Studies, in general, have identified the negative relationships between social media and vaccination intentions because social media are used more as a conduit for disseminating misinformation and negative views about vaccination and its side effects than as a conduit for delivering correct information [51,52]. The government and scientific community generally lack initiatives and activities to counter the misinformation surrounding vaccinations [51,53]. Thus, in the age of mobile phones and wireless networking, public health officials need to employ effective strategies to disseminate accurate, transparent information to the public, as well as to tailor such information to target populations that may be susceptible to misinformation on vaccination or who may be amenable to information on vaccination.

Lastly, political actors are advised to utilize political events to help gain citizens’ political trust. Studies have noted that circumstances such as events related to threats to national security can easily affect individuals’ political trust [16,54]. Indeed, contemporary episodes abound in which political trust experienced a dramatic rise or fall. The George W. Bush administration was the beneficiary of this when political trust skyrocketed under the calm leadership of President Bush in the wake of the 9/11 attack. Although the government cannot do much after political trust has fallen dramatically, occasions such as the significant jump in political trust under the Bush administration in the wake of 9/11 provided the government with abundant opportunities to enact its policies. For instance, it is difficult to deny that the quick turnaround of political trust helped the Bush administration initiate and implement the No Child Left Behind Act and the Medicare Prescription Drug Modernization Act—laws that were intended to improve educational competence among children and expand prescription drug benefits for seniors [43]. These episodes demonstrate that political actors need to be alert at all times to push the right buttons when the opportunity to promote their preferred policy is presented to them. Although it is a considerable challenge for any government to improve political trust levels of citizens in a short period, implementing the proposals noted above—promoting citizen participation in political processes, maintaining the integrity of public offices, improving government performance, utilizing social media properly for delivering government messages, seizing occasional event-driven opportunities—would go a long way to foster public confidence in the government.

In addition, it should be noted that vaccine hesitancy or refusal has induced dire economic consequences around the world [52,55]. The COVID-19 pandemic has already upended the lives of hundreds of millions of people around the world. In particular, the unvaccinated, from either vaccine hesitancy or refusal, have caused significant economic costs for governments and taxpayers [52,55]. For instance, those unvaccinated are much more vulnerable to hospitalization and death than individuals who are vaccinated, placing a significant burden on healthcare systems, other patients who also demand care, and healthcare professionals [52]. Prolonged hospitalization of those who are unvaccinated, which is mostly preventable by vaccination, adds a huge cost to the economy as well as to taxpayers [52]. Being unvaccinated can also generate global consequences because COVID-19 is highly transmissible to others in other countries [52,55]. People from countries where vaccination levels are low and international tourism is commonly enjoyed can have negative economic consequences [52,55].

Finally, the present study is not without some shortcomings. First, it has been built on a one-year cross-sectional dataset from 2021. Because this is not an experiment-driven study nor a panel study, the results cannot be generalized to other populations. Second, because the data used in our model are cross-sectional, we could not examine a causal relationship between the explanatory and dependent variables. Thus, the results need to be regarded with caution. Third, our study was based on a set of data that was all collected in a similar time frame. Thus, we cannot fully ascertain that our study is free from the threats posed by a common method variance that may be present in such a dataset. Fourth, although we focused on political trust as a moderator of the link between individuals’ political ideology and their level of trust in the government to ensure vaccine safety, it is also possible that such a link can be moderated or mediated by various factors not explored in the present study. Additionally, we admit that the insights offered by our model may not be applicable to countries where the level of vaccine hesitancy is minuscule and where vaccine hesitancy is not politically motivated. For instance, vaccine hesitancy can be driven by a multitude of factors, including the fear of its unknown side effects [56,57]. Thus, we do not argue that political trust can be a cure-all for vaccine hesitancy, but it can be a policy tool for political actors in an environment where politics can play a crucial role in deterring the public from being vaccinated.

## 5. Conclusions

The outbreak of the COVID-19 pandemic has brought opposition to vaccination to the surface [5]. Although most have regarded vaccination as a necessary step against the virus, some have raised doubts about the legitimacy of government-led vaccination programs [5,58]. In particular, conservatives have been more likely to oppose vaccination than liberals because of their ideological beliefs.

Given this context, we focused on two issues. First, we examined whether political ideology is associated with individuals’ trust in the government to ensure vaccine safety. More importantly, we explored whether ideology-driven attitudes can be shaped by the level of political trust individuals possess.

The empirical results helped answer these questions. First, we found that conservatives were unlikely to trust the government to ensure vaccine safety. Second, the results confirmed that political trust can help alter individuals’ ideology-driven attitudes toward government claims to ensure vaccine safety. In general, conservatives tend not to trust the government to ensure vaccine safety, but they are willing to trust it if they possess a high level of political trust. The results showed that the outcomes of individuals’ political ideologies are subject to being altered by factors such as political trust. Thus, the political actors who are intent on vaccinating more people against various diseases should be motivated to improve the public’s level of political trust.

## Figures and Tables

**Table 1:** Descriptive Statistics.

Variables	N	Mean	SD	Min.	Max.
Trust in government to ensure vaccine safety	473	2.27	0.65	1	3
Liberal	473	0.36	0.48	0	1
Conservative	473	0.33	0.47	0	1
Political trust	473	1.55	0.55	1	3
Distrust in scientific community	473	1.54	0.62	1	3
Age	473	52.53	16.73	19	89
Female	473	0.54	0.50	0	1
White	473	0.82	0.39	0	1
Education	473	14.88	2.93	0	20
Income	473	18.51	6.05	1	26

**Table 2:** Correlations between variables.

	Variables	1	2	3	4	5	6	7	8	9	10
1	Trust in government to ensure vaccine safety	1									
2	Liberal	0.13	1								
3	Conservative	−0.16	−0.53	1							
4	Political trust	0.30	0.20	−0.20	1						
5	Distrust in scientific community	−0.36	−0.28	0.16	−0.33	1					
6	Age	0.15	−0.17	0.17	−0.10	0.04 *	1				
7	Female	−0.11	−0.04 *	−0.09 *	0.00 *	0.16	0.01 *	1			
8	White	0.02 *	−0.06 *	0.16	−0.10	−0.10	0.05 *	−0.01 *	1		
9	education	0.13	0.15	−0.03 *	0.02 *	−0.28	0.03 *	−0.17	0.09 *	1	
10	Income	0.08 *	0.08 *	0.02 *	0.03 *	−0.21	−0.02 *	−0.15	0.15	0.46	1

Note: * *p*-value greater than >0.05.

**Table 3:** Regression Results.

	Trust in Government to Ensure Vaccine Safety			
	**Model 1**		**Model 2**	
	**Coef.**	**(S.E.)**	**Coef.**	**(S.E.)**
Liberal	−0.19	0.17	−0.51	0.48
Conservative	−0.43	0.17 **	−1.46	0.49 ***
Political trust	0.45	0.14 ***	0.16	0.20
Liberal × Political trust			0.21	0.28
Conservative × Political trust			0.67	0.32 **
Distrust in scientific community	−0.64	0.13 ***	−0.62	0.13 ***
Age	0.02	0.00 ***	0.02	0.00 ***
Female	−0.20	0.14	−0.21	0.14
White	−0.12	0.17	−0.12	0.17
Education	0.01	0.03	0.01	0.03
Income	0.00	0.01	0.00	0.01
τ_1_	−0.84	0.60	−1.31	0.61
τ_2_	1.05	0.61	0.60	0.61
Log Likelihood	−390.79		−386.65	
Wald Test	88.11		92.64	
Number of Cases	473			

Note: ** *p* < 0.05, *** *p* < 0.01.

## Data Availability

The data used here are freely available at https://gss.norc.org/get-the-data.
